# A Rare Presentation of Scrotal Lymphangioma Circumscriptum: Case Report and Review of Literature

**DOI:** 10.1155/crdm/5563847

**Published:** 2026-04-12

**Authors:** Deeptara Pathak Thapa, Mohan Bhusal, Joshana Shrestha, Smriti Piya

**Affiliations:** ^1^ Department of Dermatology, Nepal Medical College, Jorpati, Kathmandu, Nepal, nmcth.edu

**Keywords:** lymphangioma, lymphangioma circumscriptum, scrotum, vascular malformation

## Abstract

Lymphangioma circumscriptum (LC) is a rare benign lymphatic malformation of deep dermis and subcutaneous layer. Although it commonly affects the trunk, axilla, thighs, and oral cavity, its appearance in the scrotum is extremely rare and can lead to considerable psychological distress, often due to concerns regarding sexually transmitted infections. We present a case of a 42‐year‐old married Nepali male who presented with multiple slow‐growing, fluid‐filled, grouped vesicular lesions on the scrotum that had gradually increased over 12 years. The diagnosis of LC was confirmed through clinical evaluation and histopathological findings. The patient was counseled regarding the condition and was referred for surgical management.

## 1. Introduction

Lymphangioma circumscriptum (LC) is a vascular malformation caused by a developmental anomaly of the lymphatic system or may be caused by acquired conditions such as infections, radiotherapy, or surgery. It accounts for approximately 4% of vascular tumors, with an incidence of 1.2–2.8 cases per 100,0000 population [1]. LC results from the saccular dilatation of lymphatic channels, which are lined by normal single‐cell lymphatic endothelium. Clinically, it presents as persistent, grouped, and translucent vesicle [2].

The most common sites for LC include the trunk, axilla, thighs, buttocks, and oral cavity [3]. Its occurrence in the genital region is extremely rare. When present, it can cause significant psychological distress due to concerns regarding sexually transmitted diseases.

We report the case of a Nepalese male with characteristic clinical, histopathological, and ultrasonographic findings of LC. After an extensive literature search, we found only a few reports of scrotal LC, and this is the second documented case of scrotal LC in Nepal.

Scrotal LC is a rare condition that can significantly affect patients’ quality of life. Early diagnosis and appropriate management are crucial for preventing complications and reducing psychological distress. This case highlights the rarity of LC in the scrotum and emphasizes the importance of increased awareness for timely diagnosis and intervention.

## 2. Case Report

A 42‐year‐old married Nepali male presented to the dermatology department with complaints of multiple slow‐growing, fluid‐filled grouped lesions on the scrotum for the past 12 years. The lesion initially appeared on the right side of the scrotum and gradually spread to the entire scrotum. They were painless, non‐itchy, and occasionally discharged as a clear fluid. There was no history of trauma, fever, or weight loss. The patient denied a history of infection, surgery, or radiation exposure. He reported having only one sexual partner who had no similar complaints. The patient had no family history of any similar condition.

**FIGURE 1:**
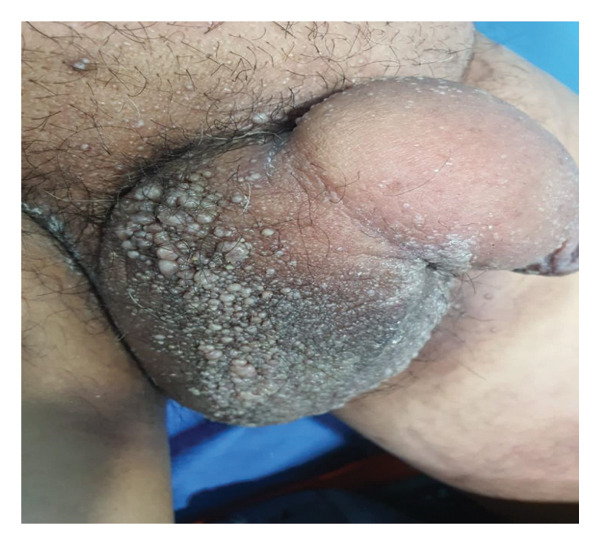
Multiple grouped vesicles in the scrotum with swelling present below the lesion extending to the shaft of the penis.

Upon examination, multiple grouped vesicles were observed on the scrotum, extending to the base of the penis and mons pubis. The underlying skin was diffusely thickened without any color change (Figure 1). Nonpitting edema extending to the penile shaft was noted. There was no lymphadenopathy or lymphedema in either lower limb.

Laboratory investigations were within normal limits, including a complete blood count, renal function test, liver function test, routine urine examination, serum lactate dehydrogenase, random blood sugar, and peripheral blood smear. Serological tests for sexually transmitted infections, including Human Immunodeficiency Virus (HIV), Hepatitis B Surface Antigen (HBsAg), Hepatitis C Virus (HCV), Treponema Pallidum Hemagglutination Assay (TPHA), Venereal Disease Research Laboratory (VDRL), and herpes simplex virus were all nonreactive.

Histopathological examination revealed dilated lymphatic channel with a collection of eosinophilic lymphatic material extending from the papillary dermis to the reticular dermis communicating with superficial vessels (Figure 2(a)) and thin‐walled lymphatic space (Figure 2(b)). Immunohistochemistry could not be performed due to financial constraints.

FIGURE 2(a) Hematoxylin and eosin stain at 4X showing multiple dilated vascular structures in the dermis. (b) Hematoxylin and eosin stain at 40X showing thin‐walled, dilated vascular lymphatic space.

**Figure:**
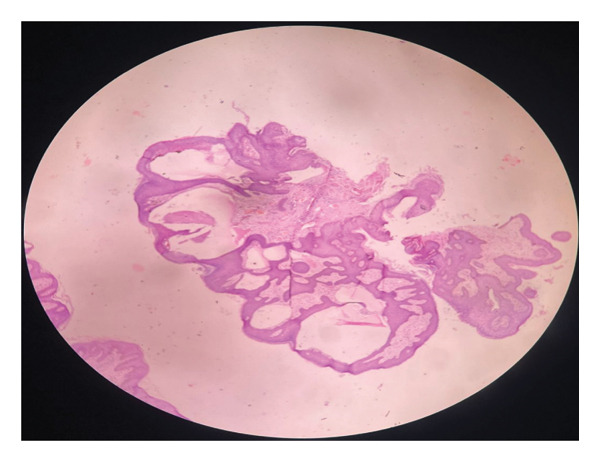
(a)

**Figure:**
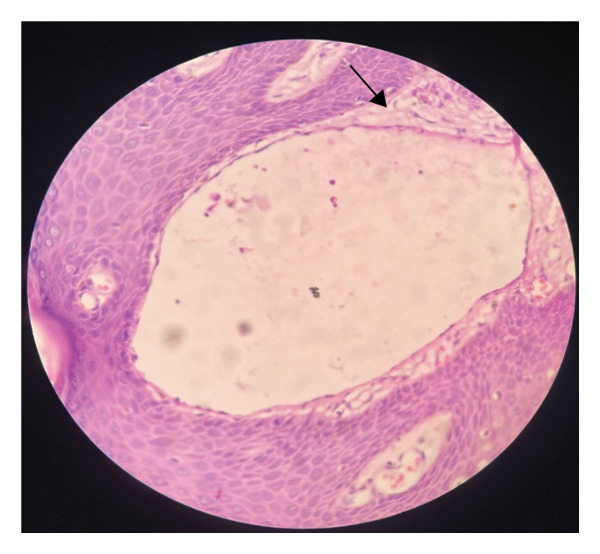
(b)

LC was diagnosed, and the patient was counseled about the condition. He was referred to the surgical department for further management.

## 3. Discussion

LC is the most common superficial lymphangioma. Fox and Fox first described the condition in 1879, referring to it as a lymphangiode. Morris introduced the term LC in 1889 [4].

LC can result from a somatic mutation and activation of the PIK3CA gene or disruption of the deep lymphatic channels due to surgery or radiotherapy [2, 5]. It has been associated with various syndromes, including Turner, Proteus, Klippel–Trenaunay–Weber, and CLOVES syndromes [5].

Although LC is primarily congenital, multiple cases of acquired LC have been reported. Acquired LC may develop due to injury to deep‐collecting lymphatics caused by infections (filariasis, lymphogranuloma venereum, tuberculosis, and erysipelas), radiation, or surgical procedures [3]. A study done by Khadka et al. reported a case of acquired scrotal LC occurring 20 years after surgery for hydrocele. However, our patient had no history of any of these predisposing conditions [6].

LC can develop anywhere on the skin and mucous membranes, with common sites including the axillary folds, shoulders, neck, proximal limbs, tongue, vulva, and buccal mucus membranes [3]. A clinicopathological study by Fatima et al. demonstrated that LC most frequently occurs in the anal/perianal area (24%), followed by the extremities (17%), tongue (14%), and vulva (10%), with the least frequent sites being the scrotum, trunk, and head and neck region (7% each) [7].

Clinically, LC is characterized by translucent or hazy vesicles of varying sizes, often grouped in a pattern resembling that of a frog spawn [1]. Dermoscopic examination typically reveals two patterns: yellow lacunae surrounded by pale septa and reddish‐blue lacunae with pale septae [8]. Differential diagnosis includes infectious diseases, such as molluscum contagiosum and filariasis. A definitive diagnosis was established through biopsy, which showed hyperkeratosis, acanthosis, and marked dilatation of lymphatic channels filled with eosinophilic infiltrates [2]. Notably, there was no endothelial swelling indicative of lymphangitis, granulomatous reaction, or the presence of adult filarial worms, which are characteristics of filariasis [9].

Lymphatic fluid discharge (lymphorrhoea) is the most common complication of laparoscopic cholecystectomy (lymphorrhoea). Other complications include ulceration, infection, pain, and regional pressure. Rare complications include squamous cell carcinoma (SCC), verruciform xanthoma, and lymphangiosarcoma [10].

Various treatment modalities are available for LC, including laser therapy, sclerotherapy, cryotherapy, imiquimod cream, and surgical excision [11]. Among these, surgical excision is considered the most definitive treatment, involving the complete removal of subcutaneous cisterns and the source of vesicles [12].

## Author Contributions

Deeptara Pathak Thapa: conceptualization, project administration, resources, writing–review and editing, and final proofreading.

Mohan Bhusal: conceptualization, project administration, resources, writing–review and editing, and final proofreading.

Joshana Shrestha: conceptualization, project administration, resources, and writing–review and editing.

Smriti Piya: conceptualization, project administration, resources, writing–original draft, and final proofreading.

## Funding

No grants or support received.

## Consent

Written patient consent has been signed and collected in accordance with the journal’s patients consent policy. Corresponding author will retain the original written consent form and provide it to the publisher if requested.

## Conflicts of Interest

The authors declare no conflicts of Interest.

## Data Availability

The data that support the findings of this study are openly available in 10.6084/m9.figshare.31806679.
